# Azaspiracid Shellfish Poisoning: A Review on the Chemistry, Ecology, and Toxicology with an Emphasis on Human Health Impacts

**DOI:** 10.3390/md20080004

**Published:** 2008-05-07

**Authors:** Michael J. Twiner, Nils Rehmann, Philipp Hess, Gregory J. Doucette

**Affiliations:** 1 Marine Biotoxins Program, Center for Coastal Environmental Health and Biomolecular Research, NOAA/National Ocean Service, 219 Fort Johnson Road, Charleston SC 29412, USA; E-mail: Mike.Twiner@noaa.gov; 2 Biotoxin Chemistry, Marine Institute, Rinville, Oranmore, Ireland; E-mail: nils.rehmann@marine.ie; 3 Biotoxin Chemistry, Marine Institute, Rinville, Oranmore, Ireland; E-mail: philipp.hess@marine.ie; 4 Marine Biotoxins Program, Center for Coastal Environmental Health and Biomolecular Research, NOAA/National Ocean Service, 219 Fort Johnson Road, Charleston SC 29412, USA; E-mail: Greg.Doucette@noaa.gov

**Keywords:** azaspiracid (AZA), AZP, shellfish poisoning

## Abstract

Azaspiracids (AZA) are polyether marine toxins that accumulate in various shellfish species and have been associated with severe gastrointestinal human intoxications since 1995. This toxin class has since been reported from several countries, including Morocco and much of western Europe. A regulatory limit of 160 μg AZA/kg whole shellfish flesh was established by the EU in order to protect human health; however, in some cases, AZA concentrations far exceed the action level. Herein we discuss recent advances on the chemistry of various AZA analogs, review the ecology of AZAs, including the putative progenitor algal species, collectively interpret the *in vitro* and *in vivo* data on the toxicology of AZAs relating to human health issues, and outline the European legislature associated with AZAs.

## 1. Introduction

In 1995, there was an outbreak of human illness in the Netherlands that was associated with ingestion of contaminated shellfish originating from Killary Harbour, Ireland. Although the symptoms were typical of diarrhetic shellfish poisoning (DSP) toxins such as okadaic acid (OA) and dinophysistoxins (DTX), the levels of DSP toxins in these shellfish were well below the regulatory level. Over the next two years it was established that these shellfish were contaminated with a unique marine toxin, originally named “Killary-toxin” or KT-3 1. Shortly thereafter, the toxin was renamed to azaspiracid (AZA) to more appropriately reflect the chemical structure of this compound 1, 2. Since the original azaspiracid poisoning (AZP) event, four additional AZP events have occurred due to contaminated Irish mussels (Table 1).

Over the last decade, various analogs of AZA have been identified in shellfish of many coastal regions of western Europe, as well as NW Africa 4 and eastern Canada (M. Quilliam, pers. comm.). Although extensive study of this toxin class has been constrained by limited availability of purified material, certified reference standards of naturally produced AZA1 are now commercially available. Limits on toxin supply may be further alleviated by the recent *in vitro* total synthesis of AZA1 5. The stage is now set to move forward rapidly in improving our overall understanding of AZAs. While certain aspects of these toxins and their pharmacological effects have been summarized previously 6, 7, we feel the timing is appropriate to critically review the advances in AZA research over the last 12 years emphasizing the chemistry, ecology, toxicology and human health impacts of AZAs, and identifying critical areas for future research.

## 2. Chemistry of AZAs

### 2.1. Structure and analogs of AZA

**Figure 1:**
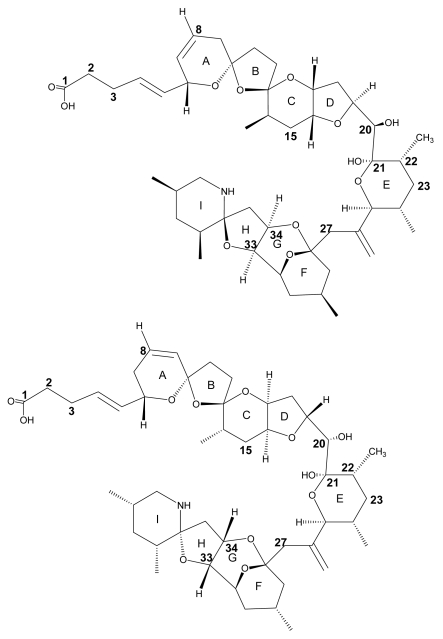
Structure of AZA1 (left) and the originally proposed structure (right). Differences between the structures are observed by the stereo-chemical orientation of rings C/D including C20, and rings F/G/H/I.

The structure of AZA1 (MW 841.5) was first reported in 1998 after successful isolation from Irish blue mussel (*Mytilus edulis*) material 2. A cyclic amine (or aza group), a unique tri-spiro-assembly and a carboxylic acid group gave rise to the name AZA-SPIR-ACID. The original structure reported in 1998 was found to contain an error, after attempts of synthesis were carried out in 2003 8, 9. The synthesised compound was found to have a different chromatographic behaviour and discrepancies in its nuclear magnetic resonance (NMR) spectrum compared to the compound isolated from natural sources. Further extensive study of the NMR spectra and more analysis resulted in structure revision in 2004 (Fig. 1) 10, 11. A detailed review of the synthetic approach and structure revision has recently been published 12.

Shortly after structure elucidation of AZA1, four additional analogs of the toxin were discovered and, after their preparative isolation, their structure was determined using MS and NMR techniques 13, 14. Three of these isolated analogs differ only in the number of methyl groups. Compared to AZA1, AZA3 is lacking the C22 positioned methyl moiety and AZA2 possesses an additional CH_3_ at position C8 (Fig. 1). The other two analogs of the toxin (AZA4 and AZA5) proved to be hydroxyl analogs of AZA3, showing the presence of an additional OH group at either C3 (AZA4) or at C23 (AZA5).

**Figure 2:**
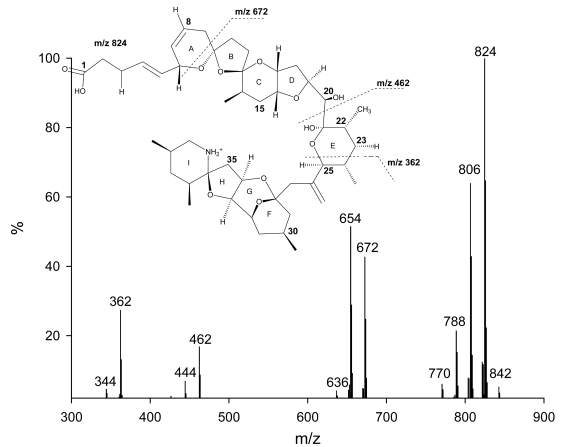
Product ion spectrum of AZA1 with significant fragmentation pattern.

So far, only AZAs 1 through 5 have been preparatively isolated, with their structure verified using NMR techniques. Structure elucidation of other analogs has been solely based on the analysis of fragmentation patterns of the respective MS/MS spectra 15–17. AZAs produce characteristic product ion spectra with four significant fragmentation groups (Figure 2) 15–18. Analysis of the different fragments has led to the identification of up to 27 different naturally occurring analogs of AZA1 as well as methyl esters of AZAs, which have been identified to be storage artefacts (Table 2) 17.

AZA6, was reported to be a positional isomer of AZA1 lacking the C22 methyl group but possessing the methyl group at C8 15, 16. In addition, hydroxy-analogs of AZA1 and AZA2 were reported 16. Very recently 12 more analogs of AZA have been reported 17. Among these analogs were dihydroxy AZAs for AZA1–3 and AZA6, as well as carboxy and carboxy-hydroxy analogs. In-depth analysis of the fragmentation pathways has shown that C23 hydroxylated AZAs produce a fragment ion at *m/z* 408 undergoing two water losses and resulting in a fragment ion at *m/z* 372. Those fragment ions are not observed with AZA analogs that do not possess an additional OH at C23. This special fragmentation pathway has aided in determining the structure of some analogs, revealing a consistent substitution of the proton at this position with a hydroxyl group.

For a number of lipophilic shellfish toxins like pectenotoxins (PTXs), OA, DTXs, brevetoxins (BTXs), and also spirolides (SPXs), fatty acid ester derivatives have been reported in shellfish tissue 19–22. Although no such esters have been identified for AZA, a variety of hydroxy-, dihydroxy- and carboxy-analogs have been discovered for AZAs (Table 2). This is not unlike analogs of YTX, where more than 80 different analogs of YTX have been reported to date 23–26 with no evidence for YTX fatty acid esters. As such, the formation of AZA toxin analogs in shellfish is similar to that of YTX with the possibility that more AZA analogs will be discovered in the future.

### 2.2. Physico-chemical properties and stability of AZAs

AZA1 was initially reported as a colourless, odourless, amorphous solid with the chemical formula C_47_H_71_NO_12_ and a molecular weight of 841.5 g/mol 1, 2. Other studies reported the toxin to be a colourless oil 5, 10. No UV absorption maxima were found above 210 nm wavelength and the refractive index of AZA1 was determined to be [α]^20^-21 (*c* 0.10, MeOH). At physiological pH, AZA1 exists as a zwitterion (i.e., contains both a positive and negative charge but is electrically neutral), which would confer detergent-like properties to this molecule 7. This overall neutral but potentially ionic character may result in enhanced possibilities for interaction of AZA with its biological target.

Little information is available about the stability of AZAs. During the production of a tissue reference material, certain techniques were tested to stabilise the tissue material for long-term storage. During a heat treatment study the toxins were observed to degrade when heated over 90 ºC 27; however, the use of gamma irradiation, which is often used to stabilise tissue reference materials, had little effect on AZA analog stability when contained in mussel matrix. Interestingly, the toxins were observed to undergo rapid degradation when irradiated as a pure compound in solution 28. AZAs stored in methanol were shown to slowly form methyl esters of the toxin 17. These esters were only observed in methanol extracts stored at room temperature or higher for prolonged periods (i.e., several months). A similar phenomenon has been shown to also occur with brevetoxin-B (PbTx-2 adduct *m*/*z* 927) 29.

Several studies have also reported the detection of AZA-like isomers that show similar or identical MS/MS spectra, but different chromatographic behaviour (Table 2) 17, 30. These isomers have not been properly characterized yet as it is first necessary to have purified material with corresponding NMR spectra in order to prove stereo-chemical differences between the compounds.

### 2.3. Isolation of AZAs

While other toxins (e.g., DTX2 or YTX) have been isolated from mussel tissue as well as phytoplankton, isolation of AZAs has only been carried out from mussel material. Although *Protoperidinium crassipes* has been identified as a potential producer of AZA 31, attempts at culturing or bulk sampling of this dinoflagellate for the purposes of toxin analysis and isolation have not yet been successful (See Section 3).

Initial isolation of AZA1 from contaminated mussel tissue consisted of extraction with acetone, liquid-liquid partitioning with hexane and 80 % aq. methanol, followed by chromatographic clean-up on silica gel, size exclusion chromatography (SEC) on Toyopearl HW-40 and ion exchange chromatography on two different materials (cationic exchanger CM650, anionic exchanger DEAE) 2. Final purification of the toxin was achieved by further chromatography on Toyopearl HW-40. To isolate AZA2 – AZA5, Ofuji *et al.* introduced further clean-up steps 13, 14. A second liquid-liquid partitioning with ethyl acetate and water helped in removing salts in addition to the hexane partitioning step. Low-pressure reverse phase chromatography on a C_18_-silica material (Develosil) resulted in a cleaner sample to be put forward to the ion exchange steps. This helped to prevent overloading of the ion exchange materials. The most crucial change of the original isolation procedure was the substitution of a final clean up on HW-40 by a reverse phase C_18_-polymeric material (ODP-50, Asahipak). Chromatography on this material facilitated the separation of the methyl and hydroxy-analogs of the toxin.

Isolation of AZA1 for production of a certified reference material (CRM) using a different extraction procedure was reported recently 32. Hepatopancreas (HP) from *M. edulis* were extracted with ethanol and partitioned with ethyl acetate and 1N NaCl solution as well as with hexane and 90% methanol. The sample was further purified using vacuum liquid chromatography on silica, SEC on Sephadex LH-20, flash chromatography on LiChroPrep RP-8, and a final purification step on a C_8_-silica column (high performance liquid chromatography; HPLC). Using a HPLC reverse phase material in the final purification step has increased purity to > 95 % as determined by NMR and liquid chromatograph-mass spectrometry (LC-MS) 33.

## 3. Azaspiracid Ecology

A pre-requisite to better understanding the ecological aspects of an algal biotoxin is the identification of the organism(s) responsible for its production. Not only does this information allow researchers to focus their work on known toxigenic organisms, but what is already known about the ecology of the causative species adds a valuable perspective on factors that may influence the production and distribution of the toxic compound. As has been the case for many of the major algal biotoxin classes, AZAs were isolated and described originally from a secondary source, namely *M. edulis*, several years after the initial poisoning outbreak 2. Despite the fact that four additional human intoxications have followed the original event in 1995 (Table 1) and AZAs have become more widespread (Table 3), now including multiple European countries as well as Morocco and eastern Canada, the identity of the toxin producer remains elusive.

There have been several attempts to identify the AZA producing organism(s) and the polyether structure of these compounds might suggest a dinoflagellate origin 2, 40, as has been demonstrated for a number of other polyether biotoxins (e.g., BTX, ciguatoxin (CTX), DTX, YTX, PTX). Most notable is the effort by James *et al.* involving the isolation by hand and extraction of 2000 cells of the heterotrophic dinoflagellate, *P. crassipes* 30, 41. These authors reported the detection by liquid chromatography-multiple tandem mass spectrometry of AZA1, 2, and 3 in the *P. crassipes* extract (equivalent to 200 cells), leading them to propose this taxon as capable of synthesizing AZAs 30. Nonetheless, while others have detected AZAs in the plankton 32, 42, follow-up investigations involving the isolation of *Protoperidinum* spp. (including *P. crassipes*) cells from field samples and analysis by mass spectrometry have yet to corroborate these earlier findings 43. In addition, cell counts of *Protoperidinium* spp. ranging from 600 to 900 cells L^−1^ have been associated with little or no AZAs in Irish mussels 44.

The ability of *P. crassipes* to accumulate measureable amounts of AZAs is not in question 30; however, the heterotrophic nature of this dinoflagellate (acknowledged by James *et al.* 2003) requires consideration of its potential role as a vector rather than progenitor of these toxins 44. Off the southwestern coast of Ireland, Gribble *et al.* 45 observed *P. crassipes* (in addition to 31 other *Protoperidinium* spp.) in abundances that could account for consumption of 30% of the standing stock of phototrophic dinoflagellates per day. In addition, Miles *et al.* 43 observed *P. crassipes* and other *Protoperidinium* spp. cells ingesting *Dinophysis* spp. in field material, likely explaining the source and consistent presence of either OA or PTX in isolated cells of the former species. It is possible that the physiological status of the *P. crassipes* cells isolated by James *et al.* was optimal for toxin production. In fact, it is well-established that algal biotoxin levels associated with other known toxigenic species can vary from high to undetectable in field populations, likely in response to various environmental factors 46. Moreover, differences in toxin production among *Protoperidinium* spp. might not be unexpected given the moderate to high degree of LSU rDNA sequence variability (i.e., base pair substitutions, insertions, deletions) between and within species of this genus. 47. Although several investigators have maintained *P. crassipes* in laboratory cultures, attempts to confirm production of AZAs and manipulating environmental variables to determine their effects on growth and toxicity were not successful. Such an impediment was experienced for several decades with the OA/DTX producers *Dinophysis* spp., until the elegant work of Park *et al.* 48 revealed a complex trophic relationship with the kleptoplastidic ciliate *Myrionecta rubra* and its cryptophyte prey *Teleaulax* sp., which provided the key to survival and growth in culture of *D. acuminata*. Similar insights may be required to elucidate the appropriate culture conditions for growth of putative AZA producers/accumulators. It is certainly feasible that AZAs or their biosynthetic intermediates are synthesized by multiple species of protists or even bacteria, both of which can serve as prey for heterotrophic or mixotrophic taxa such as *Protoperidinium* and other dinoflagellates 43, 49, 50. Indeed, bacteria and cyanobacteria are known producers of a diverse array of polyether compounds 51, 52 that could include the structural building block(s) of AZAs.

Although the true source of AZAs awaits confirmation, analysis of plankton samples has provided information on the suite of analogs potentially available to shellfish and other organisms in adjacent trophic compartments. In addition to the isolation and extraction of single cells outlined above, an approach employing mesh bags containing a synthetic resin to adsorb both particulate and dissolved algal biotoxins has been used to obtain samples from the water column 53, 54. Bags/disks of exposed resin or resin-containing cartridges 53 through which water has been pumped (enabling large volumes to be concentrated; C. Miles, pers. comm.) are then extracted and analyzed for AZA content and composition. To date, only AZA1, 2, and 3 have been detected in the plankton/water column and in all cases the toxin composition was dominated by AZA1, comprising at least 80 percent of total AZAs 30, 32, 42, 55. Together, these findings indicate that the remaining AZA analogs (see Table 2) isolated from shellfish are likely produced once the former three analogues are incorporated into the digestive tract and tissues of these organisms; however, the mechanism(s) by which such bioconversions occur (e.g., physico-chemical vs. enzymatic processes) remain to be determined. Given the recent commercial availability of an AZA1 CRM 56 and the anticipated production of CRMs for AZA2 and 3, studies of AZA bioconversions by specific shellfish tissues or isolated enzymes are now possible and will provide further insight as to the natural origin of other AZA derivatives. In addition, once identification and/or laboratory culture of the AZA-producing organism(s) are achieved, studies can be conducted to further assess *in vivo* biotransformations, as reported for other algal biotoxins such as DSP and paralytic shellfish poisoning (PSP) toxins 57. Such experiments should also be used to begin examining the potential for direct physiological effects of AZAs on species known to accumulate these toxins, about which little is currently known.

Much useful information can be gained by examining the spatio-temporal relationship between biotoxin profiles of the toxigenic organisms and those in vectoring species (e.g., shellfish). Once the extent and kinetics of biotransformations are established for individual vectors following exposure to plankton-derived toxins, it may be possible to determine the toxin source (for no/minimal conversions) or potentially estimate the timing and duration of a bloom event (for more extensive toxin metabolism) as for the PSP toxins 58. Azaspiracid toxin profiles have been examined in both a single algal species 30 and in bulk plankton material 32, 42, with AZA1 clearly predominant in all cases and AZA2 detected in far lower amounts. However, while AZA3 was present in isolated cells (< 10%), it was not detected consistently in bulk samples, suggesting either a different toxin source organism(s), another strain(s) of the same species with a different intrinsic toxin composition, or an environmental modulation of toxin composition, none of which can be confirmed or refuted at this time. The fact that AZA3 did not occur in the bulk plankton sampled 32, 42, yet was found in co-occurring mussels, suggests that this analog may also be produced via biotransformation in bivalves.

From a seasonal perspective, it appears that AZA contamination of shellfish above the EU regulatory action limit (> 0.16 μg/g) has been detected between mid-summer and mid-winter from northern/western European waters 33, 35, 59 to its present, southern-most extent in Morocco (July, ~0.9 μg/g) 4. In certain cases, the presence of AZAs in phytoplankton does correspond to the timing of shellfish contamination, yet toxin levels in bivalves can remain elevated for eight to twelve months following the initial exposure 7, 55, lasting into the winter months when conditions are generally unfavorable for phytoplankton growth. In the case of mussels, this protracted contamination was suggested to result from the movement of AZAs from their initial location in the HP into other tissues, where depuration occurred more slowly 7, 35, 36, 55, 59–61, but more recently, the presence of a putative AZA-binding protein(s) that may prolong retention time in shellfish has been studied. Specifically, a 45 kDa protein of unknown identity has been isolated from the HP of AZA contaminated *M. edulis* that binds to AZA 62. Alternatively, the prolonged retention of AZAs during winter months may at least in part be explained through reduced metabolic activity of shellfish during periods with reduced temperatures. However, the continued or sporadic presence of AZA-producing species as a *de novo* toxin source during this prolonged “depuration phase” can not be discounted 33. It should also be noted that the DSP toxins (i.e., OA, DTXs) have been reported to co-occur with AZAs in shellfish during some years but not others 32, suggesting that the organisms producing these two toxin classes may share certain growth requirements or seasonal distributions, but are likely distinct taxa.

Bioconversion of algal toxins once taken up from the plankton by vector species can heavily influence net toxicity due to the often disparate intrinsic potencies of individual toxin derivatives. However, there are relatively scant comparative toxicity data available for the respective AZA analogs (see Section 4.2.1.). Published estimates based on mouse i.p. administration indicate that AZA2 and 3 are slightly more potent than AZA1 1, 14 and that AZA4 and AZA5 are less potent 13. The range of bivalve species in which AZAs have been detected includes mussels (*M. edulis*, original 1995 outbreak; *M. galloprovincialis*), oysters (*Crassostrea gigas, Ostrea edulis*), scallops (*Pecten maximus*), clams (*Tapes philipinarum, Ensis siliqua*, *Donax* spp.), and cockles (*Cerastoderma edule*) (Table 3) 33, 36, 38, 59, 63. Note that AZAs have also been found recently in crustaceans such as crabs (*Cancer pagurus*) 39.

In all shellfish examined to date, either AZA1 or AZA2 represents the majority of total AZAs, with AZA3 generally at minimal levels or absent altogether 4, 7, 33, 55, 59–61. Interestingly, the occurrence of AZA3 at concentrations representing > 5% of total AZAs has thus far been restricted to mussels, suggesting the possibility of shellfish species-specific biotransformations as have been reported for other biotoxins such as PSP toxins 64. Toxin profiles of AZAs reported thus far are generally similar for a given shellfish type regardless of the geographic location. For example, profiles in Spanish mussels (*M. galloprovincialis*) are comparable to those found in both Irish and Norwegian mussels (*M. edulis*), as is the case for scallops (*P. maximus*) from both France and Ireland 35, 36, 60. An exception to this trend was reported recently by Taleb *et al.* in their analysis of mussel (*M. galloprovincialis*) digestive glands from Morocco, which exhibited the apparent dominance of AZA2 (75–100%) instead of AZA1 4. Again, it is not currently possible to ascribe this finding to either toxin metabolism within the shellfish (e.g., methylation of AZA1), the toxin composition of the planktonic source organism, or to evaluate the possible influence of extensive sample clean-up and concentration steps. In terms of tissue distribution of AZAs, some studies show a variable but clearly predominant localization of toxin in the digestive gland versus whole flesh (average, ~5-fold; range, ~2.5- to 10-fold) 65, whereas others report the presence of toxin primarily in tissues other than the HP (64–100%) 61. Dissection techniques of small organs such as the HP in raw mussels are difficult, and can lead to cross contamination of other organs, which may in turn explain some of the discrepancies found between the authors. It has been found in scallops that fluids containing domoic acid (DA) from the HP can “stain” (i.e., contaminate) other tissues very consistently even through further washing steps 66.

The implications of the preceding discussion relate primarily to predicting the potential risk to humans from consuming shellfish contaminated with AZAs. However, this group of toxins also has the potential to exert negative impacts within marine foodwebs. A recent study by Colman *et al.* demonstrated the teratogenic effects of AZA1 on embryos of the Japanese medaka, *Oryzias latipes*, a widely used *in vitro* fish model 67. Drastic changes in the developmental process caused by AZA-1 indicate that ingestion of this toxin by wild fish (or potentially other marine organisms) could decrease the survival rate of hatchlings and possibly cause adverse effects at the population level, in cases of widespread and appropriately timed exposure. Critical evaluation of this possibility will require confirmation of the toxin source organism, the factors influencing toxin production, and elucidation of routes of AZA trophic transfer.

## 4. Toxicology of Azaspiracids

### 4.1. Human toxicity

Unlike many of the other well-described marine phycotoxins, relatively little is known about AZA. Similar to DSP toxins, human consumption of AZA-contaminated shellfish can result in severe acute symptoms that include nausea, vomiting, diarrhea, and stomach cramps. Due to the limited data available from many of the AZP events, nearly all information regarding AZA toxicology has been obtained from controlled *in vitro* and *in vivo* experiments. Many of these efforts have been directed towards assessing the risk of AZA consumption in contaminated shellfish and in turn, identifying the molecular target(s) of AZA, which is currently unknown.

### 4.2. In vivo toxicology

#### 4.2.1. Mouse bioassay and intraperitoneal injection

During the initial AZP intoxication event in 1995 involving shellfish from Killary Harbour, Ireland, mussel extracts tested highly positive in the DSP rat bioassay and the DSP mouse bioassay 68. However, in the absence of significant levels of OA or DTX2, and mouse symptomology atypical of DSP or YTX toxins 1, 69, AZA1 was subsequently identified in these samples 2. In mice, intraperitoneally (IP) injected acetone extracts of contaminated mussels caused “neurotoxin-like” symptoms characterized by sluggishness, respiratory difficulties, spasms, progressive paralysis, and death within 20–90 minutes 1, 68, 70. The IP minimum lethal dose of partially purified AZA (i.e., KT-3) was 150 μg/kg 1. IP injection of a lethal dose (>150 μg/kg) caused swelling of the stomach and liver concurrent with reduction in size/weight of the thymus and spleen 71. There was vacuole formation and fatty acid accumulation in the hepatocytes, parenchymal cell pyknosis in the pancreas, dead lymphocyte debris in the thymus and spleen, and erosion and bleeding in the stomach. The pathological changes induced by AZA were stated to be unique from those induced by DSP, PSP and amnesic shellfish poisoning (ASP) toxins.

The IP minimum lethal dose of AZA2 (8-methylazaspiracid) and AZA3 (22-demethyl-azaspiracid) were 110 and 140 μg/kg, respectively 14, suggesting higher potency relative to AZA1. However, AZA4 and AZA5 (hydroxylated versions of AZA3; see Table 2) are less potent with lethal dose values of 470 and < 1000 μg/kg, respectively 13. Assuming equivalent degrees of purity, the order of AZA analog potency by IP injection appears to be AZA2 > AZA3 > AZA1 > AZA4 > AZA5. However, future studies should further corroborate these findings through sufficiently replicated and controlled LD_50_ (lethal dose, 50%) determinations using toxins of known purity.

#### 4.2.2. Acute oral administration

Crude extracts of AZA (> 900 μg/kg) were given orally to mice via gastric intubation 71. At six fold the IP injected dose that induced 100% mortality, all mice survived with no clinical signs after 24 h. However, mice autopsied at 4 h displayed various gastrointestinal (GI) perturbations such as accumulation of fluid from the ileum, and necrosis of epithelial cells on the microvilli. At 8 h these effects were further exacerbated, but at 24 h, with the exception of fused microvilli, were predominantly absent. These observations are strikingly similar to many chemically-induced models of inflammatory bowel disease (IBD) such as Crohn’s disease and ulcerative colitis 72.

**Figure 3:**
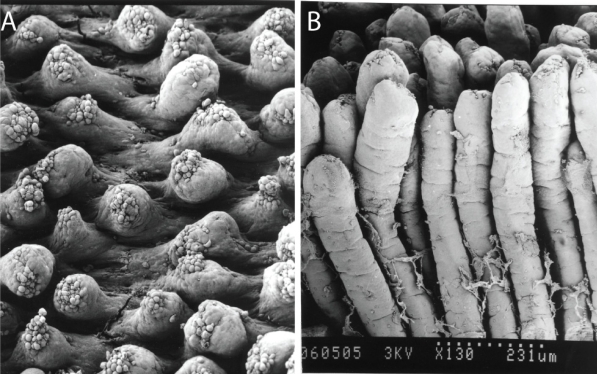
Scanning electron micrographs of mouse small intestinal villi (A) at 8 h following a 700 μg/kg acute oral dose of AZA1 and (B) undamaged villi for comparative purposes. Photos courtesy of Dr. Emiko Ito, Chiba University, Japan.

Subsequent studies using purified material have further detailed the effects of AZA1 on mice 73. Male ICR mice were orally administered single doses of AZA1 ranging from 300 to 900 μg/kg for up to 24 h. All mice receiving 900 μg AZA1/kg were sacrificed prior to 24 h. Although there was not a clear dose-response, likely due to insufficient experimental replicates, an approximate oral minimum lethal dose with purified AZA1 was 500 μg/kg. Lower doses of 300 μg/kg induced fatty acid droplet accumulation in the liver as soon as 1 h following intubation, followed by sporadic degeneration and erosion of the small intestinal microvilli, vacuole degeneration in epithelial cells, and atrophy of the lamina propria at 4 h. Mid-level doses of AZA1 (500 to 700 μg/kg) revealed progressive intestinal erosion at 8 h and continued atrophy of the lamina propria at 24 h (see Figure 3). However, at 24 h there were fewer degenerating epithelial cells in the microvilli suggesting some signs of recovery. By 24 h, the liver had increased in weight by 38% in the 500 μg/kg dose group. There were time- and dose-dependent effects on the number of necrotic lymphocytes in the thymus, spleen, and the Peyer’s patches of the small intestine, which was elegantly supported by quantification of the number of non-granulocytes (lymphocytes, monocytes, macrophages) in the spleen. AZA treatments of 600 and 700 μg/kg resulted in a 33% decrease in the number of non-granulocytes, which were primarily T and B lymphocytes. There were no reported histological changes associated with the kidney, heart, and lung, and unfortunately, brain tissue was not examined. Compared to mice orally exposed to OA, the damage elicited by AZA was slower in onset with much longer times required for recovery 73. Many of these same effects were also observed in mice treated with synthesized AZA1, but with slightly reduced potency (i.e., minimum lethal dose >700 μg/kg vs. ~500 μg/kg with natural AZA1) 74. Unfortunately, percent purities were not reported. In addition, the diastereomer C_1_-C_20_-*epi*-AZA1 had at least a 3- to 4-fold reduction in *in vivo* potency (*ca.* > 3000 μg/kg) with virtually no morphological effects on the GI system, thus suggesting that stereo-specific orientation of the molecule is important for induction of enhanced toxicity. This finding may also corroborate some of the *in vivo* differences between the crude extracts and the purified toxin. Twelve truncated AZA1 analogs, produced during the total synthesis of AZA1, did not elicit any signs of toxicity suggesting that correct orientation and full length of the molecule are required for full potency. It is also suspected that since the effects elicited by AZA are not localized to only the GI system, that AZA1 and/or its metabolites can be absorbed from the GI system and can be at least partially distributed to various organ systems (P. Hess, unpubl. data). Due to limited toxin supplies, there are no data available on the potency of any additional AZA analogs (i.e., AZA2, 3) via oral administration. As such, future studies are necessary to determine the LD_50_ values for at least AZA1, 2, and 3.

#### 4.2.3. Repeated oral administration

In another series of *in vivo* exposure studies, mice were orally administered repeated doses of AZA1 and then monitored for recovery 75. Severe injuries were induced by two repeated doses of 250, 300, 350, or 450 μg/kg, two days apart, and recovery was monitored for up to 90 days. Of the 16 mice receiving 450 μg/kg, 11 died prior to the second dose, suggesting a revised minimum oral lethal dose of < 450 μg/kg. In fact some mice died at 250 and 300 μg/kg, but only two replicate mice were available for each dose. Pathological effects were similar to those reported in above during acute oral administrations and IBD in human and murine models. Recovery times for each tissue were: liver = 7 days; lymphoid = 10 days; lung = 56 days, and the stomach = >12 weeks. During this time there were also signs of bacterial infection in the stomach lining. Although these findings of protracted recovery times are likely a function of tissue-specific turnover time and the rates of AZA1 metabolism and elimination, they correspond with the *in vitro* findings of the irreversible nature of AZA1 induced cytotoxicity 76 (see Section 4.3). In a separate set of experiments, mice were orally administered up to 40 repeated, low doses of AZA1 (1 to 50 μg/kg) over a period of 145 days (*ca*. 2 gavages per week) 75. Ten percent of the mice survived the 40 repeated injections at the highest dose (50 μg/kg), as the other 90% were sacrificed due to extreme weakness, and 30% of the mice in the 20 μg/kg treatment group were also sacrificed early. Reductions in the weight of various tissues (heart, liver, kidney, spleen, thymus) appeared to be manifested by up to a 35% whole body weight loss. These effects were likely due to reduced nutrient absorption in the eroded GI tract. Similar to the effects of higher oral AZA1 doses (> 250 μg/kg), the GI tract displayed obvious signs of erosion (i.e., edema, shortened and damaged microvilli) and an accumulation of gas. As well, there were typical effects on the spleen and thymus. However, low dose repeated exposures of AZA1 caused mild liver inflammation with virtually no effects on liver fatty acid content. Lung tissue displayed signs of interstitial inflammation and bleeding. Although not observed in a dose-dependent manner, there was a low incidence of lung tumor formation (20% incidence; 4 out of 20 mice in the 20 and 50 μg/kg treatment groups) and hyperplasia (enlargement of the tissue due to accumulation of cells) in the stomach (60% incidence; 6 out of 10 mice in the 20 μg/kg treatment group). Lung tumors were only observed after 2–3 months into the recovery phase and were S-100 reactive. S-100 proteins were originally thought to be of neurogenic origin but are now known to be common among many cancerous and inflamed tissues 77. Brain tissue was not analyzed in these experiments. Interestingly, microarray studies using T lymphocytes have identified an S-100 gene as being differentially expressed following exposure to AZA1 78. Repeated treatments of mice with 1 or 5 μg/kg displayed significant GI effects suggesting that the lowest observable adverse effect level (LOAEL) for AZA1 is on the order of 1 μg/kg in mice, which is comparable with the LOAELs estimated in humans of *ca.* 0.4 to 2 μg/kg bodyweight in the two risk assessments that were conducted by the Food Safety Authority of Ireland (FSAI) 3, 79.

### 4.3. In vitro toxicology

#### 4.3.1. Effects on protein phosphatase activity

Due to the similarities in GI symptoms that AZAs have in common with OA and DTXs, AZAs were originally classified together with the DSP toxins. It was first postulated, with good reasoning, that the most likely mechanism of action, similar to OA, was protein phosphatase (PP) inhibition 80. PPs are well described regulators of cell signaling pathways where they act in a manner opposite to that of kinases by removing phosphate groups from proteins. The serine/threonine PPs are known to be inhibited by OA 81. The effects of crude blue mussel extracts containing AZAs demonstrated no indication of PP1 enzyme inhibition 82 and a subsequent study utilizing the same assay format but with PP2A, also found no effects of purified AZA1 on enzyme activity 83. However, we cannot exclude the possibility that AZA may still inhibit one of the many other types of serine/threonine PPs (i.e., PP2B, PP2C, PP4, PP5) or another PP sub-type (i.e., tyrosine-specific phosphatase, lipid phosphatase). In fact, OA and genistein (a tyrosine kinase inhibitor) have been shown to modulate the cytosolic calcium response induced by AZA1 in human lymphocytes 84.

#### 4.3.2. Cytotoxicity

On their own, many phycotoxins (i.e., CTX, BTX, saxitoxin) are not known for inducing cell death. The original observation that AZAs can cause cytotoxicity were performed by Flanagan *et al.* 69, 80, 82 using HepG2 hepatoblastoma cells and human bladder carcinoma cells (ECV-304) exposed to crude mussel extracts. Additional studies have since confirmed these findings, in a time- and concentration-dependent manner, for a variety of other cell types from various mammalian sources (Table 4).

The only cell type that has been tested so far that is not sensitive to AZA1-induced cytotoxicity appears to be the human colon Caco-2 cells. This is in contrast to the effects of AZA1 on intestinal epithelial cells *in vivo* as observed following oral intubation in mice (see Section 4.2.). Caco-2 cells are often used as a model for studying intestinal drug transport 89 and/or transepithelial electrical resistance (TEER) 90. However, these assays require a standard 17 to 21 day growth period to allow for cell growth, monolayer formation, and cellular differentiation. It is unclear if the Caco-2 cells listed in Table 4 were allowed to grow to these densities prior to the addition of AZA1, and if so, what effect this might have on observation of a cytotoxic response. At high cell densities, the viability detection substrate (i.e., Alamar blue, MTT) used in some cytotoxicity determinations will become limited. Nonetheless, monolayers of Caco-2 cells exposed to AZA1 have shown significant reductions in TEER assays when exposed to low concentrations of AZA1 (i.e., 5 nM) 31, 86. Visual confirmation has demonstrated monolayer perturbations that result in a loss of electrical resistance across the epithelial cells suggesting that even if the Caco-2 cells are not susceptible to an AZA1-induced cytotoxic response, they are sensitive to the effects of AZA1 on monolayer integrity. These observations are more in line with the *in vivo* observations of Ito *et al.* 73–75 (see Section 4.2.).

During the cytotoxicity experiments listed in Table 4, a variety of morphological effects were observed. In T lymphocytes, cells initially responded to AZA1 by a reduction in membrane integrity, organelle protrusion concurrent with flattening of cells, and a retraction of their pseudopodia or lamellipodia 83. This was followed by protracted cell lysis. Although caspase-3 was not induced during these exposures (M. Twiner, unpubl. observ.), this alone does not rule out apoptotic cytotoxicity. During these experiments, filamentous actin (i.e., F actin) was also monitored and observed to closely follow the retraction of the pseudopodia. Although actin levels were not quantified, it appeared as though F actin was reorganized following AZA1 exposure. Isolated enzyme assays investigating rates of F actin polymerization and depolymerization suggest no direct effect of AZA1 on this protein (M. Twiner, unpubl. observ.). Experiments using human breast cancer cells and mouse fibroblasts exposed to AZA1 also illustrate reductions in cellular proliferation and density that are similar to the action elicited by YTX 85. However, F-actin measurements indicated no change following 1 nM AZA1 exposure for 24 h.

AZA1 and an enantiomer of AZA1 induced distinguishable morphological and cytoskeletal (i.e., F actin) effects on human neuroblastoma cells following 24 – 48 h exposures 76, 91. Low concentrations of AZA1 (10 nM) appear to induce retraction of the neurites (cellular projections) and cell rounding with simultaneous actin cytoskeleton disarrangement. Although previous studies by these authors have shown quantitatively decreased levels of F actin following AZA1 exposure, the concentrations of AZA1 required to induce these effects were very high (~7.5 μM) 84. Interestingly, the effects of AZA1 on morphological, cytoskeletal, and cell viability appear to be irreversible 76, 88, 91, which may explain the protracted *in vivo* rate of GI recovery following mouse intubations (see Section 4.2.).

DSP toxins such OA are well-described inducers of apoptosis 92. Initial studies suggested that AZA1 does not induce an apoptotic response, but rather induces necrotic lysis. These observations were made based on cytotoxic morphological observations 83, the absence of mitochondrial membrane potential changes in neuroblastoma cells 84, and the absence of caspase-3 induction in T lymphocytes (M. Twiner, unpubl. observ.). However, more recent evidence suggests that AZA1 induces apoptosis. This is not surprising as none of the previously observed characteristics are definitive indicators of apoptosis. In the same neuroblastoma cells used previously to monitor the effects of AZA1 on mitochondrial membrane potential, caspases were subsequently shown to be activated as determined using caspase-specific fluorescently tagged peptides 91. However, these data do not lend insight into which caspase subtype(s) (i.e., caspase-1, -3, -7, -9, etc.) is/are up regulated as the peptide sequence used in these experiments binds a broad-spectrum of caspases. These data of caspase activation are further supported by a recent *in vivo* study in mice where dead or dying lymphocyte cells in the spleen and thymus were observed to be undergoing pyknosis - chromatin condensation indicative of apoptosis 74. Clearly, more efforts are needed to define the effects of AZAs on the apoptotic pathway.

#### 4.3.3. Intracellular signaling molecules

Many AZA analogs have been shown to cause a variety of effects on intracellular signaling molecules. In mammalian cells, cytosolic calcium is an important secondary messenger for a variety of pathways, including cell death 93, 94 and many marine toxins are known to modulate cytosolic calcium 95–99. Human lymphocytes exposed to AZA1 (200 nM) were shown to elevate cytosolic calcium levels by *ca*. 50% above basal 84. This response was shown to be sensitive to extracellular calcium, PKC (protein kinase C) activation, PP inhibition, and cAMP (cyclic adenosine monophosphate) elevation. In addition, elevations in cAMP were sensitive to adenylate cyclase inhibition but insensitive to PP inhibition and extracellular calcium. cAMP is a second messenger that responds to membrane receptor activation and often functions to activate kinases. Similarly, AZA2 and AZA3 also elevated cytosolic calcium and cAMP 100, whereas AZA4 did not affect basal cytosolic calcium levels but did have an inhibitory effect on calcium uptake from the extracellular medium 101. Differences in the effects of the various AZA analogs may be a function of solubility and/or purity as it would be highly unusual for a class of structurally related compounds (with only single methyl and/or hydroxyl substitutions) to elicit completely different mechanisms of action rather than, more commonly, various degrees of affinity and efficacy. Although these studies were unable to identify a specific mechanism of action, the modulation of cytosolic calcium and cAMP may be influenced by modulation of a membrane protein.

#### 4.3.4. Membrane proteins

In addition to the growing body of literature on the effects of AZAs on cellular actin cytoskeleton and intracellular signaling pathways, there are many new and novel insights being generated by investigations into the effects of AZAs on membrane proteins such as claudins and cadherins. Claudins are integral membrane proteins involved in tight junction cell adhesion and are pivotal in paracellular transport of epithelial and endothelial cells 102 with at least 24 known claudin types 103. Caco-2 epithelial cells exposed to AZA1 demonstrated an increase in soluble and insoluble fractions of claudin-2 protein expression and a decrease in insoluble claudin-3 31. These responses appeared to be reversibly mediated by ERK 1,2, members of the mitogen activated protein kinase (MAPK) family of proteins that commonly respond to extracellular stressors or signals 104. Epithelial-cadherins, or E-cadherins, are transmembrane Adherens proteins that are involved in cell-to-cell adhesion 103. In Caco-2 cells, a fragment representing an extracellular domain of E-cadherin was up regulated following exposure to AZA1 85. And recently, it was shown that T lymphocytes exposed to AZA1 up regulated gene expression and protein levels of low density lipoprotein receptor (LDLR) by as much as 3.5- and 2.5-fold, respectively 78. This LDLR effect appears to be in response to decreased levels of intracellular cholesterol caused by AZA1. LDLR is a well described membrane protein that endocytoses cholesterol bound to low density lipoproteins into the cell. Cholesterol is required to maintain membrane integrity and function and is intricately involved with tight junction and Adherens proteins 105. Although premature to determine decisively, yet consistent with currently available *in vitro* data, AZA1 appears to be targeting a membrane protein such as a claudin, cadherin, or LDLR, in turn eliciting an effect on intracellular signaling molecules and the cytoskeleton, ultimately resulting in cytotoxicity. These cellular effects are also in line with the *in vivo* pathologies and potential carcinogenicity or tumor promoting capabilities of this toxin.

## 5. Human health issues

### 5.1. Acute illness

As mentioned in the Toxicology sections, all symptoms observed in humans following consumption of shellfish contaminated with AZAs appear within hours of ingestion, and include nausea, vomiting, severe diarrhea and stomach cramps 68. The illness persists for 2–3 days and full recovery has been established in all cases during the incident in Arranmore Island, 1997 3. To date, no long term effects or illness have been reported.

AZP remains a rare illness, as only 5 intoxication events have been reported to date (Table 1). Due to the similarity with ‘food poisoning’ or DSP, it has to be assumed that more cases exist; however, it is likely that there is a high percentage of under-reporting due to the symptoms of the illness disappearing rapidly (i.e., days) and no precedence for fatality.

### 5.2. Risk of chronic effects

Given that shellfish production sites remain open for production if AZA contamination levels are below the EU regulatory limit (160 μg/kg shellfish meat), a large number of shellfish consignments with low levels of AZAs have been produced in Ireland since 2001, and subsequently marketed and consumed in Europe.

Some chronic illnesses are caused by chemicals that are retained in the human body for a long time. A prime example for such a chronic condition is cancer, which may be caused through the accumulation of carcinogens that may be absorbed with fatty foods, transferred to the human body through the blood stream and accumulate in all fatty tissues of the body. Therefore, by analogy, in order to evaluate whether AZAs have a potential for causing chronic effects, it is important to know whether AZAs, or their metabolic products, are distributed to the body via the blood stream, or whether they are simply excreted with food after exerting their toxic action in the intestine. Although pilot studies with mice have shown increased occurrence of cancer and hyperplasia with a protracted rate of recovery in the test animals exposed to AZA1 75, this increase in cancer was not observed in a dose-dependent manner (1 case at highest dose of 50 μg/kg body weight, 3 cases at the intermediate dose of 20 μg/kg body weight). Also, the number of mice used in this study was relatively low (due to a lack of toxin) and the results may only be considered indicative, suggesting the need for further studies to elucidate the carcinogenic potential of AZAs. In addition, other toxic effects were observed in mice exposed to AZA over 20 weeks 75, particularly in the highest exposure dose, therefore, chronic effects cannot be excluded at this stage. Damage caused by AZA to epithelial cells of the intestinal tract *in vivo* 73–75, were supported *in vitro* by Hess *et al.* 106, providing a model to explain the diarrhetic symptoms in humans. It is conceivable that AZAs may contribute to chronic disorders in the GI tract, such as IBD (i.e., Crohn’s disease, ulcerative colitis), or cancers of the stomach, intestines and colon. However, it remains to be established whether the suspected bioavailability and toxicity of AZAs are similar when dosed as part of naturally contaminated shellfish as food.

To date, due to the scarcity of epidemiological data, it has not been possible to establish any link between AZAs and the occurrence of cancer or birth defects in humans. Any studies presuming consumption of AZA-contaminated shellfish and statistically comparing a hypothetically increased occurrence of chronic disease with levels of AZA in shellfish would be convoluted by the multitude of potential carcinogenic agents and contaminants of any consumer area. Therefore, it is the opinion of the authors that research in this area should focus on the toxicokinetics (i.e., absorption, metabolism, distribution, and elimination) of AZAs in animals to establish the possible fate of AZAs in the human body, until larger amounts of synthesized AZA or AZA-contaminated shellfish are available facilitating statistically valid long-term exposure trials. Also, when the mode of action is clarified from *in vitro* studies, further studies may be necessary to evaluate chronic effects, depending on the mechanism elucidated.

### 5.3. Possible synergistic effects of AZAs with other shellfish toxins

A number of different toxin groups may co-occur in shellfish, and thus the question arises as to whether these toxins would interact with each other to suppress or enhance their respective toxicities, or whether the different toxic effects are simply independent of each other. No observations have been reported in humans to date on any synergistic effects of different phycotoxin groups. This area of investigation is a relatively new direction in toxicology. While guidelines have been discussed since 1999 107, the UK Food Standards Agency (FSA) has published a draft action plan on risk assessment of mixtures of pesticides and similar substances 108. Similarly, the US EPA has issued a document on a strategy for risk assessment of the cumulative effects of co-occurring pesticides 109; however, no such risk assessments are currently available for shellfish toxins.

While co-occurrence of the OA-group and PTXs are essentially pre-programmed due to production of both groups by *Dinophysis* spp., the co-occurrence of other toxins is determined by overlapping autecology and toxigenic potential of their algal producers. As quantitative data on the occurrence of AZAs in shellfish has been mostly reported from Ireland and Norway, the information on possible combinations is very limited. In Ireland, several co-occurrences of AZAs with toxins from the OA-group have been observed during 2001, 2005, 2006 and 2007 (Marine Institute, unpubl. monitoring data). On one occasion, the appearance of AZAs and OA/DTX2 in mussels (*M. edulis*) was simultaneous in mid-August 2001 at Bantry Bay, Southwest Ireland 33, and depuration of both toxin groups followed a similar rate. Subsequent analysis of these shellfish also showed that PTXs had been present in these shellfish at the same time 20. Therefore, it must be assumed that co-occurrence of at least these 3 toxin groups is possible. In Norway, the co-occurrence of AZAs with YTXs may be a problem, as both toxin groups have been observed in this region, in addition to analogs of SPX, PTX and OA 110.

Since AZAs and OA-group toxins both initially target the GI tract leading to acute GI disorders, possible interactions of these toxin groups are of obvious concern. Although the effects of AZA and OA in the intestine of mice are unique, both at molecular and at microscopic levels, both toxins appear to target the microvilli 73–75. OA transiently caused shortened microvilli, whereas AZA caused distinct and protracted microvilli erosion. By analogy, both toxins separately cause diarrhea in humans after consumption of contaminated shellfish. Therefore, it seems very likely that the effects of combined AZAs and OA-group toxins would at least be additive, if not synergistic. However, to date, no information is available from human epidemiology, and no animal experiments have been conducted in this area.

Synergistic toxicity of AZAs and YTXs is a further concern. With the exception of some effects on mouse cardiac muscle cells 111, YTXs have not been explicitly involved in human poisoning and have not shown any toxicity when orally administered to mice 112, 113. Nonetheless, their toxic potential is still significant as estimated from IP injection in mice (LD_50_ = 100–700 μg/kg bodyweight) 113, 114. Therefore, it could be postulated that the toxicity of YTXs may be of greater importance to human health if they co-occur with AZAs. During such co-occurrence, AZAs could affect the permeability of the intestinal barrier through an eroded epithelial or GI lining, and YTXs may be able to enter the human blood stream and subsequently cause damage that they otherwise could not exert.

### 5.4. Safe levels of AZAs in shellfish and allowable daily intake

Considering the lack of knowledge on the long term exposure potential of AZAs with the fact that all other shellfish toxins have only been evaluated for their acute toxic potential, experts consulted during the *ad hoc* consultation by FAO/IOC/WHO 115 advised regulators to consider only known acute effects in humans when establishing safe levels of AZAs in shellfish. Allowable daily intakes require the evaluation of repeated intake and a risk from long term exposure. As long term effects have not been shown to date in humans, the establishment of allowable daily intakes is not appropriate. Therefore, a proposed regulatory level of AZAs in shellfish has only been obtained through estimation of the consumption of a single portion of shellfish and through the estimation of an Acute Reference Dose (ARfD).

Analogous to other formal risk assessments, the risk assessment by FSAI also used epidemiology data to establish the ARfD 3. This risk assessment was based on a statistical approach, which makes use of known distributions of all components involved in a poisoning event (i.e., mussel weight, portion size consumed, concentration of AZAs in mussels, etc.), and the result is a distribution of most likely ARfDs. It is then the responsibility of risk managers and regulators to adapt an ARfD from this distribution (e.g., the most probable value or the ARfD covering with 95% probability all possible ARfDs). The selected ARfD is then used, in combination with an appropriate safety factor, to derive a No Observable Adverse Effect Level (NOAEL). This statistical approach used for risk assessment of AZAs is novel and has not been used by the FAO/IOC/WHO expert group; however, as cited in the risk evaluation by FSAI, “*it is the opinion of expert toxicologists that a more accurate estimate may be obtained using this method*” 115.

The FSAI 2006 risk assessment was the second risk assessment by this group, following the first one in 2001 79. The reason for review of the earlier risk assessment was the availability of new scientific data allowing a re-estimation of the amount of toxin consumed by the people who fell ill during the 1997 Arranmore AZP incident (Table 1), which is still the best documented incident to date. The new evidence mostly related to new information on the tissue distribution of AZAs in mussels (*M. edulis*), the effects of heat-treating mussels on their toxin content and the ratios of AZA analogs observed in shellfish tissues 65. The median, or most likely ARfD derived in this assessment was 0.63 μg/kg body weight, while the most likely LOAEL was divided by a safety factor of 3. When assuming consumption of a 250 g portion of shellfish flesh and a human average weight of 60 kg, this ARfD translates into a proposed regulatory level of AZAs in shellfish flesh equal to 160 μg/kg. Co-incidentally, this is the same level as was transposed into EU legislation in 2004 (853/2004/EC) 116. This limit of 160 μg AZA/kg shellfish in flesh has also been proposed by the EU Commission for Health and Consumer Protection (EU-DG Sanco) to *Codex Alimentarius* as part of a draft Codex standard on fish and fisheries products during recent meetings of Codex 117, 118. As this limit had also been implemented in EU legislation since 2002 (2002/225/EC) 119, although now superseded by 853/2004/EC 116, it had been implemented in Ireland since 2002. Surveillance information from the Marine Institute, the Irish National Reference Laboratory (NRL) as cited in Anon. (2006) 3, suggests that numerous lots of shellfish have been produced since 2002 that were contaminated at levels close to this concentration. Although there is a high likelihood of under-reporting of illnesses due to the rapid recovery, it has been estimated by the authors that millions of portions of shellfish (several thousand tons) with low but significant AZA-levels have been consumed since 2001 (contamination range of 100 – 160 μg/kg). Therefore, assuming a reporting rate of only 0.1 %, there should have been thousands of reports of illness if the current limit of 160 μg/kg was unsafe. However, not a single report was received on any poisoning incident from these shellfish, over the period from 2001 to 2007. Therefore, it can be assumed that existing risk management practise has shown - in retrospect - that this level is indeed a safe level protecting consumers from acute effects of AZAs in shellfish.

It should be noted that this limit has been derived from epidemiological observations caused by a mixture of naturally occurring analogs. As outlined in the section on Chemistry of AZAs, it is clear that at least 28 analogs may occur in shellfish. The limit has been derived from consideration of the three main analogs only (AZA1, 2 and 3). As shown by Satake *et al.* 2, and Ofuji *et al.* 13, 14, AZA4 and 5 have a lower toxicity in mice when injected via IP, suggesting a decreased toxic potential for oxidized AZA analogs, a very common structure-activity relationship. All other analogs 15–17 occur at very low concentrations and are even further oxidised, suggesting that their toxic potential is reduced from both the structure-activity relationship established by Satake *et al.* and Ofuji *et al.* and from their concentration. Additionally, no information was available from the 1997 Arranmore AZP incident on these analogs, and therefore, their consideration would have been very difficult.

### 5.5. Regulatory limits in the EU and implementation approaches

The first regulatory limit established explicitly for AZAs was 100 μg/kg shellfish meat, and was introduced in Ireland in 2001 as a consequence of the risk assessment by Anderson *et al.* 79. However, this limit was only introduced as a working practice in the management of Irish shellfish production, and was seriously flawed by the lack of tools available for implementation of this limit. Although the mouse bioassay for lipophilic toxins 21 had never been officially validated for detection of any shellfish toxin, it was clear from studies by Satake *et al.* 2 and Ofuji *et al.* 120 that the test was not likely to detect AZAs at this level, therefore ruling out use of the reference test prescribed by European legislation at the time 121. Only one other method was available during this period (i.e., LC-MS); however, this method had also not been officially validated and, as no AZA standards were internationally available, validation efforts were severely hampered.

The EU Commission for Consumer Health and Protection (DG Sanco) held a workshop in Brussels in 2001 and reviewed available information 122, leading to the introduction of a provisional limit for AZAs of 160 μg/kg shellfish meat 119. Although, the risk evaluators initially proposed a safe limit of 80 μg/kg, it was believed at the time that the reference test (MBA) would be able to only implement a level of 160 μg/kg. More recent considerations suggest that the MBA for lipophilic toxins only has, at best, a 50% chance of detecting toxins at this level and is not appropriate to effectively manage shellfish production. Nevertheless, the regulatory limit was based on the LD_50_ of the MBA, demonstrating that food safety regulations can only be as effective as the methods available at the time. This unsatisfactory situation had generated significant discussion in the working groups of the EU NRLs and the Community Reference Laboratory (CRL). In an initial attempt to improve the situation, the EU Commission responded through a call for research projects on the validation of alternative (i.e., non-animal based) methods for all lipophilic toxins problematic in the EU, including OA/DTXs, PTXs, YTXs, and AZAs. In late 2004, the EU funded three projects in this area, BIOTOX (www.biotox.org), BIOTOXmarin (www.biotoxmarin.de) and DETECTOX (www.detectox.eu), all due to finish in 2007. In the meantime, the expert consultation by FAO/IOC/WHO 115 has recommended the official validation of LC-MS as a reference test for many lipophilic toxins, including AZAs. Additionally, it is a legal obligation for the EU CRL to lead the NRLs in method development and validation 123. Thus, in 2005, DG Sanco has also explicitly tasked the EU-CRL to collaborate with the NRLs on the validation of LC-MS methods as alternative tools to replace the current MBA reference test. Furthermore, a workshop between DG Sanco and the European Centre for the Validation of Alternative Methods (ECVAM) recommended reducing and replacing the MBA for lipophilic toxins as rapidly as possible due to its technical limitations and ethical concerns 124. In anticipation of a positive outcome of these efforts, DG Sanco has also transposed these regulations into a more recent updated version of European Food Safety Legislation 116, 125, 126.

Recent progress and difficulties in the preparation of adequate quality control tools in the development, validation and implementation of analytical methods for shellfish toxins has been reviewed by Hess *et al.* 26. Previous work by the authors in collaboration with the National Research Council-Canada (NRC-Canada) has led to the production of a CRM for AZA1 that is now commercially available worldwide 31, 56. The same project (Azaspiracid Standards and Toxicology; ASTOX), funded under the Irish National Development Plan 2000–2006, also led to significantly improved understanding on the preparation of homogenous and stable reference shellfish tissues for lipophilic toxins, including AZAs 27, 127, 128; some of these materials have also been used in proficiency testing via a scheme for Quality Assurance in Marine Environmental Matrices in Europe (QUASIMEME, 2006–2007). Finally, the ASTOX project, in collaboration with the NRCC and the Institute for Reference Materials and Measurement (IRMM), has also yielded two candidate certified reference shellfish tissues, one mussel material contaminated predominantly with AZAs, the other one a mussel material contaminated with a number of toxins groups, including OA/DTXs, AZAs, YTX, PTX2, SPX and others. Therefore, significant progress has been made in the preparation of QC tools for the quantitative determination of AZAs in shellfish tissues. Further work in this area should include the certification of the candidate reference tissues and the preparation of reference standards for AZA2 and 3, two analogs that appear also of toxicological importance and that have been explicitly regulated in the EU 129.

## 6. Conclusions and future directions

In less than a decade since the identification of AZA1 in Irish shellfish, much progress has been made in the field of AZA research and its application to protecting the health of seafood consumers. Most convincing is the fact that although there were five AZP events between 1995 and 2000, since that time (over 7 years) there have not been any further AZP events due to accidental ingestion; a testament to the regulatory mechanisms in place and not for lack of AZA in shellfish over this period. This is clearly a case of where advances in research knowledge have aided various shellfish monitoring programs, contributed to the implementation of safe regulatory limits, and ensured compliance by the aquaculture industry. Nonetheless, AZA research remains in its infancy. Major research efforts are required to identify the causative organism(s), which will subsequently be useful in determining the environmental and genetic cues that influence or trigger the production of AZAs. Monitoring programs will also benefit as there is often, but not always, a correlation between the presence of potentially toxigenic phytoplankton species and the subsequent accumulation of toxins in shellfish. The impacts of AZAs on shellfish fecundity and viability are only anecdotal and require well controlled *in vitro* and mesocosm studies for these effects to be determined. With the exception of AZA accumulation in various species of shellfish, routes of trophic transfer through the food web are completely unknown, although AZAs have recently been found in crustaceans. AZA could have serious impacts on the fishery industry as the teratogenic effects of AZA1 have been documented in fish embryos.

Until recently, LC-MS methodologies for the detection of AZAs were hindered by the lack of an AZA CRM. Although AZA1 is currently available through the NRC-Canada, there is no commercial source for some of the other important, and potentially more toxic, AZA analogs (i.e., AZA2 and 3). Other detection methods such as an ELISA (enzyme-linked immunosorbent assay) have recently been developed for AZA 130 but are not yet commercially available. Relative to LC-MS detection, ELISAs are economical, high-throughput, often have very low detection limits, and require less training to operate, making them very suitable for many end users such as researchers and aquaculture managers. A supply of a readily available AZA ELISAs would allow for major advancements in the field of AZA research.

Much progress has been made in the areas of AZA toxicology and associated human health issues, but similar to the other areas of AZA research, efforts are impeded by the lack of purified toxin standards and high-throughput detection methods. AZA1 administered to mice via IP injection and oral intubation is highly toxic with similar lethal doses. Analogous to DTX1 114, the *in vivo* potencies of AZA do not appear to be related to the route of administration. These findings are in contrast to microcystin-LR 131, palytoxin 132, and YTX 114 whose oral potencies in mice are at least 1–2 orders of magnitude lower than IP injection.

Definitive mechanism of action studies are still necessary and currently nothing is known regarding mixture toxicities, either with other AZA analogs or other toxin classes (i.e., OA, DTXs, DA, PTX, saxitoxin), that can also be found in shellfish in many of the same regions where AZAs occur. There is a lack of information regarding AZA toxicokinetics (i.e., absorption, distribution, metabolism, elimination), well characterized epidemiology studies, and detailed information on the carcinogenic or tumor promoting potential of AZAs. Given that azaspriacids are now being reported from the coastal waters of an increasing number of countries on a global scale, it is clearly imperative to advance our understanding of AZA toxicity and ability to rapidly detect these toxins and their progenitor(s) organisms. Addressing these and other issues outlined above will be among the aims of researchers, regulators, and public health officials over the years ahead.

## Figures and Tables

**Table 1:** Reported cases of azaspiracid poisoning (AZP) 1995–2007^3^.

Location of AZP	Date	Implicated food source	Amount consumed	Area of production	Number of illnesses recorded
Netherlands	November 1995	Mussels (*Mytilus edulis)*	Not recorded	Killary Harbour, Ireland	8
Ireland	September /October 1997	Mussels (*M. edulis)*	“As few as 10–12 mussels”	Arranmore Island, Ireland	Estimated 20–24 (8 seen by a doctor)
Italy	September 1998	Mussels (*M. edulis)*	Not recorded	Clew Bay, Ireland	10
France	September 1998	Scallops (*Pecten maximus*)	Not recorded	Bantry Bay, Ireland	Estimated 20–30
United Kingdom	August 2000	Frozen mussels (*M. edulis)*	Not recorded	Bantry Bay, Ireland	12–16

**Table 2:** Overview of all reported AZA analogs.

Abbrev.	Original analog	Substituent	Name	Ref.
AZA1			Azaspiracid	2
AZA2			8-methyl-azaspiracid	14
AZA3			22-desmethyl-azaspiracid	14
AZA4	AZA3	OH	22-desmethyl-3-hydroxy-azaspiracid	13
AZA5	AZA3	OH	22-desmethyl-23-hydroxy-azaspiracid	13
AZA6			22-desmethyl-8-methyl-azaspiracid	15, 16
AZA7	AZA1	OH	3-hydroxy-azaspiracid	16
AZA8	AZA1	OH	23-hydroxy-azaspiracid	16
AZA9	AZA6	OH	22-desmethyl-3-hydroxy-8-methyl-azaspiracid	16
AZA10	AZA6	OH	22-desmethyl-23-hydroxy-8-methyl-azaspiracid	16
AZA11	AZA2	OH	3-hydroxy-8-methyl-azaspiracid	16
AZA12	AZA2	OH	23-hydroxy-8-methyl-azaspiracid	16, 17
AZA13	AZA3	2 OH	22-desmethyl-3,23-dihydroxy-azaspiracid	17
AZA14	AZA1	2 OH	3,23-dihydroxy-azaspiracid	17
AZA15	AZA6	2 OH	22-desmethyl-3,23-dihydroxy-8-methyl-azaspiracid	17
AZA16	AZA2	2 OH	3,23-dihydroxy-8-methyl-azaspiracid	17
AZA17	AZA3	COOH	carboxy-22-desmethyl-azaspiracid	15, 17
AZA18	AZA1	COOH	carboxy-azaspiracid	17
AZA19	AZA6	COOH	carboxy-22-desmethyl-8-methyl-azaspiracid	17
AZA20	AZA2	COOH	carboxy-8-methyl-azaspiracid	17
AZA21	AZA3	COOH + OH	carboxy-22-desmethyl-3-hydroxy-azaspiracid	17
AZA22	AZA1	COOH + OH	carboxy-3-hydroxy-azaspiracid	17
AZA23	AZA6	COOH + OH	carboxy-22-desmethyl-3-hydroxy-8-methyl-azaspiracid	17
AZA24	AZA2	COOH + OH	carboxy-3-hydroxy-8-methyl-azaspiracid	17
AZA25	AZA3	-H_2_O	21-22-dehydro-22-desmethyl-azaspiracid	17
AZA26	AZA1	-H_2_O	21-22-dehydro-azaspiracid	17
AZA27	AZA6	-H_2_O	21-22-dehydro-22-desmethyl-8-methyl-azaspiracid	17
AZA28	AZA2	-H_2_O	21-22-dehydro-8-methyl-azaspiracid	17
AZA29	AZA3	COOCH_3_	22-desmethyl-azaspiracid-1-methyl-ester	17
AZA30	AZA1	COOCH_3_	Azaspiracid-1-methyl-ester	17
AZA31	AZA6	COOCH_3_	22-desmethyl-8-methyl-azaspiracid-1-methyl-ester	17
AZA32	AZA2	COOCH_3_	8-methyl-azaspiracid-1-methyl-ester	17

**Table 3:** Azaspiracid analysis in marine shellfish and crustaceans.

Country	Region / City	Year(s) observed	Organism	Maximum conc. in whole flesh (μg/g)^a^	Ref.
Ireland	West coast	1995, 1997, 1999, 2000, 2001, 2005, 2006, 2007	*Mytilus edulis,Crassostrea gigas,Ostrea edulis,Pecten maximus, Tapes philipinarum, Ensis siliqua, Cerastoderma edule*	8.0	7, 33
Norway	SW coast / Sognefjord	1998	*M. edulis*	0.82^b^	34, 35
England	E coast / Craster	1998	*M. edulis*	0.13^b^	35
Spain	Galicia	2001	*M. galloprovincialis*	0.24	36
France	Brittany	2001	*P. maximus*	0.80	36
Denmark		2002	*M. edulis*	<LOQ	37
Portugal	Southern coast	2003	*M. galloprovinciali*s, *Donax* spp.	0.016	38
Morocco	NW coast	2004	*M. galloprovincialis*	0.9	4
Canada	Nova Scotia	2005	*M. edulis*	<LOQ	M. Quilliam, pers. comm.
Sweden	Tjärno	2005	*Cancer pagurus*	0.019^c^	39
Norway	North coast	2005, 2006	*Cancer pagurus*	0.049^d^	39

a Concentrations less than 0.01 μg/g are typically below the limit of quantification (LOQ) and should only be seen as indicative of presence.

b HP value. Total tissue was not available.

c Highest value detected in HP. No AZAs were detected in the white meat (claws + body).

d Values up to 0.733 μg/g were observed in the HP.

**Table 4:** Overview of azaspiracid cytotoxicity.

Cell type	Cell line	Source	Cytotoxic	EC_50_ (nM) a	Method of analysis b	Ref.
B lymphocyte	Raji	human	yes	1.6 *	MTT	83
breast cancer	MCF-7	human	yes	> 1 *	DNA content; cell number	85
Colon adrenocarcinoma	Caco-2	human	no	nd	DNA content	85
Colon adrenocarcinoma	Caco-2	human	no	nd	Alamar blue	31, 86
Embryonic kidney	HEK-293	human	yes	4.6 *	MTT	83
Hepatoblastoma	HepG2	human	yes	unknown	MTT	69, 80, 82
Lung epithelial	A547	human	yes	1.5 *	MTT	83
Monocyte	THP-1	human	yes	2.4 *	MTT	83
Neuroblastoma	BE(2)-M17	human	yes	n.d.	morphological	76
T lymphocyte	Jurkat E6-1	human	yes	1.1	G6PH	78
T lymphocyte	Jurkat E6-1	human	yes	3.5	MTT	83
Bladder carcinoma	ECV-304	human	yes	unknown	MTT	69, 80, 82
cerebellar granule cells	primary	mouse	yes	0.87	MTT	87
cerebellar granule cells	primary	mouse	yes	n.d.	LDH	87
Neuroblastoma	Neuro-2A	mouse	yes	2.3 *	MTT	83
Skin fibroblasts	primary	mouse	yes	> 10	DNA content	85
Spinal cord neurons	primary	mouse	yes	n.d.	Action potentials	88
Pituitary epithelial	GH_4_C1	rat	yes	16.8	MTT	83

a Effective concentration that results in 50% cell death at 24 h. Values designated with an asterisk (*) are EC_50_ values at 48 h. Note: nd = values not determined and “unknown” indicates cytotoxicity induced by crude mussel extracts.

b MTT, MTS, and Alamar blue are mitochondrial enzyme-dependent viability assays. G6PH (glucose-6-phosphate dehydrogenase) and LDH (lactose dehydrogenase) assays are based on the release of these cytosolic enzymes from intact cells.

## Abbreviations

### Abbreviations

ARfD: acute reference dose
ASP: amnesic shellfish poisoning
ASTOX: Azaspiracid Standards and Toxicology
AZA: azaspiracid
AZP: azaspiracid shellfish poisoning
BTX: brevetoxins
cAMP: cyclic adenosine monophosphate
CRL: community reference laboratory
CRM: certified reference material
DA: domoic acid
DSP: diarrhetic shellfish poisoning
DTX: dinophysistoxin
ECVAM: European Centre for the Validation of Alternative Methods
ELISA: enzyme-linked immunosorbent assay
EU-DG Sanco: EU Commission for Health and Consumer Protection
FSA: Food Standards Agency
FSAI: Food Safety Authority of Ireland
G6PH: glucose-6-phosphate dehydrogenase
GI: gastrointestinal
HP: hepatopancreas
HPLC: high performance liquid chromatography
IBD: inflammatory bowel disease
IP: intraperitoneal
IRMM: Institute for Reference Materials and Measurement
KT: Killary-toxin
LC-MS: liquid chromatograph-mass spectrometry
LD_50_: lethal dose, 50%
LDH: lactose dehydrogenase
LDLR: low density lipoprotein receptor
LOAEL: lowest observable adverse effect level
LOQ: limit of quantification
MAPK: mitogen activated protein kinase
MTS: 3-(4, 5-dimethylthiazol-2-yl)-5-(3-carboxymethoxyphenyl)-2-(4-sulfophenyl)-2H-tetrazolium
MTT: 3-(4, 5-dimethylthiazolyl-2)-2, 5-diphenyltetrazolium bromide
NMR: nuclear magnet resonance
NOAEL: no observable adverse effect level
NRC-Canada: National Research Council Canada
NRL: National Reference Laboratory
OA: okadaic acid
PKC: protein kinase C
PP: protein phosphatase
PSP: paralytic shellfish poisoning
PTX: pectenotoxin
QUASIMEME: Quality Assurance in Marine Environmental Matrices in Europe
SEC: size exclusion chromatography
SPX: spirolides
TEER: transepithelial electrical resistance
YTX: yessotoxins
